# Unraveling the Potential Role of Glutathione in Multiple Forms of Cell Death in Cancer Therapy

**DOI:** 10.1155/2019/3150145

**Published:** 2019-06-10

**Authors:** Huanhuan Lv, Chenxiao Zhen, Junyu Liu, Pengfei Yang, Lijiang Hu, Peng Shang

**Affiliations:** ^1^School of Life Sciences, Northwestern Polytechnical University, Xi'an, Shaanxi 710072, China; ^2^Research & Development Institute of Northwestern Polytechnical University in Shenzhen, Shenzhen 518057, China; ^3^Zhejiang Heye Health Technology Co. Ltd., Anji, Zhejiang 313300, China; ^4^Research Centre of Microfluidic Chip for Health Care and Environmental Monitoring, Yangtze River Delta Research Institute of Northwestern Polytechnical University in Taicang, Suzhou, Jiangsu 215400, China; ^5^Key Laboratory for Space Bioscience and Biotechnology, Northwestern Polytechnical University, Xi'an, Shaanxi 710072, China

## Abstract

Glutathione is the principal intracellular antioxidant buffer against oxidative stress and mainly exists in the forms of reduced glutathione (GSH) and oxidized glutathione (GSSG). The processes of glutathione synthesis, transport, utilization, and metabolism are tightly controlled to maintain intracellular glutathione homeostasis and redox balance. As for cancer cells, they exhibit a greater ROS level than normal cells in order to meet the enhanced metabolism and vicious proliferation; meanwhile, they also have to develop an increased antioxidant defense system to cope with the higher oxidant state. Growing numbers of studies have implicated that altering the glutathione antioxidant system is associated with multiple forms of programmed cell death in cancer cells. In this review, we firstly focus on glutathione homeostasis from the perspectives of glutathione synthesis, distribution, transportation, and metabolism. Then, we discuss the function of glutathione in the antioxidant process. Afterwards, we also summarize the recent advance in the understanding of the mechanism by which glutathione plays a key role in multiple forms of programmed cell death, including apoptosis, necroptosis, ferroptosis, and autophagy. Finally, we highlight the glutathione-targeting therapeutic approaches toward cancers. A comprehensive review on the glutathione homeostasis and the role of glutathione depletion in programmed cell death provide insight into the redox-based research concerning cancer therapeutics.

## 1. Introduction

Glutathione is a thiol-containing tripeptide consisting of L-glutamate, cysteine, and glycine [1]. It is abundantly distributed in mammalian cells and mainly exists in the forms of reduced glutathione (GSH) and oxidized glutathione (glutathione disulfide (GSSG)). GSH is predominately distributed in the cytosol and to a lesser content in the subcellular organelles, such as the mitochondria, nucleus, and endoplasmic reticulum (ER). GSH takes part in many cellular metabolic activities including reactive oxygen species (ROS) removal, DNA and protein syntheses, and signal transduction [2, 3].

As for cancer cells, they need a greater ROS level than normal cells for the enhanced metabolism and vicious proliferation [4, 5]. Nevertheless, the higher ROS level can also be counteracted by an increased activity of the antioxidant defense system which copes with the higher oxidant state. The GSH system is one of the major cellular antioxidant systems that cooperatively maintain and synergize the redox balance [6]. The increased GSH level has been observed in different human cancer cells and is an important contributor to cancer pathology and the resistance to anticancer therapy [7]. As a contrary, GSH depletion increases the susceptibility of cancer cells to various forms of programmed cell death and sensitivity to chemotherapies [8]. Consequently, the role of GSH in the initiation of programmed cell death in cancer cells has been well implicated in accumulative studies. There are crosstalks and interrelationships between these different forms of programmed cell death induced by GSH.

Here, we highlight the GSH homeostasis, the relationship between GSH and oxidative stress, the recent findings of GSH depletion in multiple forms of programmed cell death, and GSH-targeting therapeutic approaches toward cancers. The review may help to better understand the role of GSH modulation in cell death and shed light on the possibility of finding new therapeutic approaches based on the redox system for cancers.

## 2. GSH Homeostasis

### 2.1. GSH Synthesis

**Figure 1:**
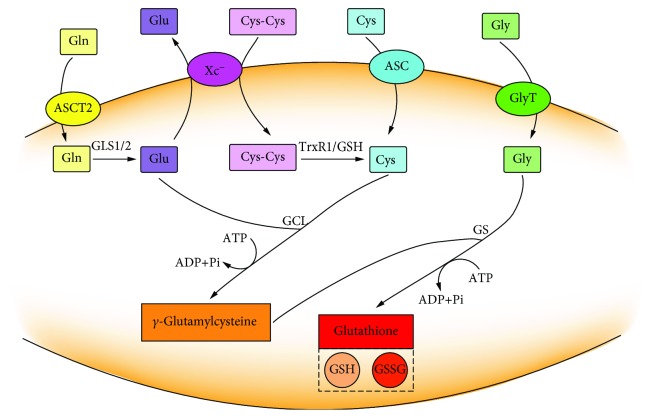
Two-step enzymatic reaction of glutathione synthesis. The first step is the coupling of L-glutamate and cysteine to produce *γ*-glutamylcysteine under the catalysis of glutamate-cysteine ligase (GCL). The second step is the coupling of *γ*-glutamylcysteine to glycine catalyzing by glutathione synthetase (GS). Each reaction consumes one ATP molecule. Glutathione exists in the forms of reduced GSH and oxidized GSSG.

The biosynthesis of glutathione was obtained by catalyzing of L-glutamate, cysteine, and glycine through continuous two-step enzymatic reactions which depend on ATP [9]. Glutamine is hydrolyzed by glutaminase (GLS1/2) to form glutamate after being absorbed into the cell via a transmembrane amino acid transporter (ASCT2). Cysteine can be directly absorbed by an amino acid transporter (ASC) or can be obtained by reduction of cystine absorbed by system X_c_^−^. The intracellular glycine can be directly absorbed by a glycine transporter (GlyT). The synthesis of glutathione is through two-step enzymatic reactions by glutamate-cysteine ligase (GCL) and glutathione synthetase (GS) (Figure 1). In the first step, GCL catalyzes the reaction of cysteine with glutamate to produce *γ*-glutamylcysteine; next step, *γ*-glutamylcysteine is combined with glycine to produce glutathione under the catalysis of GS [10]. Since the concentration of *γ*-glutamylcysteine is negligible when GS is present, GCL determines the rate of GSH synthesis during this process [11].

Glutathione exists in the reduced GSH form and oxidized GSSG form. The content of glutathione is in a dynamic balance through the regulation of synthesis, utilization, metabolism, and efflux. Under physiological condition, GSH is the predominant form which is more than 98%, while GSSG is less than 1% [12].

### 2.2. GSH Distribution

**Figure 2:**
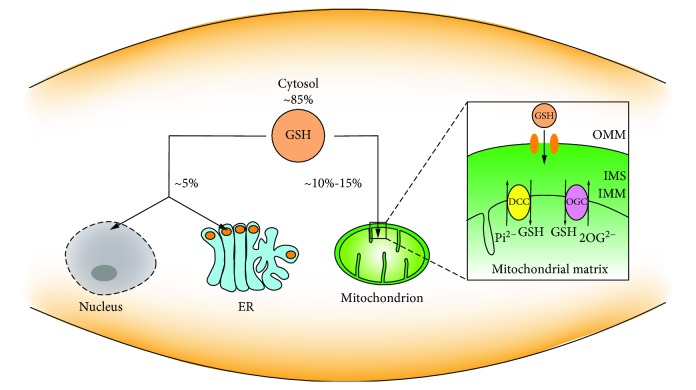
Distribution of intracellular GSH. GSH is distributed in the cytosol, nucleus, mitochondria, and ER.

The glutathione-centered redox system participates in the redox signal network and controls cell growth, development, and oxidant defense [13]. In addition to the cytoplasm, glutathione also presents in various subcellular organelles, including the nucleus, mitochondria, and ER (Figure 2). There is a significant difference in glutathione distribution among these subcellular organelles [14, 15]. The distribution of glutathione in different intervals is critical because it establishes a redox environment that supports various metabolic and signaling events [16]. The maintenance of redox homeostasis of the nucleus, mitochondria, ER, and other organelles as well as the extracellular environment is inseparable from glutathione.

#### 2.2.1. Cytosolic GSH

In mammalian cells, glutathione is exclusively synthesized in the cytosol and about 85% of it remains where it was synthesized [17, 18]. In the cytosol, glutathione is mainly in the reduced form. The ratio of GSH : GSSG in the cytosol is conservatively estimated at about 10000 : 1~50000 : 1 [19]. Reports show that the concentration of the cytosolic GSH is as high as 10 mM, while GSSG in the cytosol is as low as nanomolar concentration. The redox potential of E_GSH_ in the cytosol is about 320 mV [20]. The highly reduced GSH pool has also been found in a variety of species [21, 22]. The cytosol contains the largest GSH pool, which does not contradict its distribution of GSH in other subcellular organelles. Due to the lack of GS in subcellular compartmentation, GSH must be imported into the subcellular organelles from the cytosol.

#### 2.2.2. Mitochondrial GSH

Mitochondria are coated by two membranes and separated into two spaces, the matrix surrounded by the inner mitochondrial membrane (IMM) and the intermembrane space (IMS) between the IMM and the outer mitochondrial membrane (OMM). Although the enzymes in these two separate chambers, the IMS and matrix, are not identical, each is providing NADPH and exchanging molecules through its mechanism. Mitochondria are the main sites for aerobic respiration and producing ROS, mainly O_2_^-·^. Mn-dependent superoxide dismutase (MnSOD) reduces O_2_^-·^ to H_2_O_2_, and the gradual accumulation of H_2_O_2_ further generates free radicals. In the mitochondria, catalase reduces H_2_O_2_ to H_2_O and O_2_ but due to the low catalase content, a certain amount of GSH is required to maintain the redox balance. During the oxidation of GSH to GSSG by glutathione peroxidase (GPX), H_2_O_2_ is reduced to H_2_O, which can offset the H_2_O_2_ produced by MnSOD [23, 24].

The mitochondrial glutathione (mGSH) pool only accounts for 10%~15% of the total glutathione pool, and the internal glutathione is mainly present in a reduced state [25, 26]. Considering the mitochondrial volume, the concentration of mGSH per mitochondria is similar to that of cytosolic GSH and there is no concentration gradient in the mitochondrial inner membrane space. Mitochondria are not able to synthesize GSH as for lacking GS, but they can take up GSH from the cytosol [23]. GSH in the cytosol passes through the two layers of the OMM and IMM to reach the destination in the mitochondria. The monotonous uptake of GSH through the OMM is facilitated by the pore proteins, which allow molecules less than ~5 kDa to freely pass [27]. The concentration of small molecules in the IMS is equivalent to the concentration in the outer cytoplasm. Small molecules entering the IMS cannot penetrate into the mitochondrial matrix because of the different lipid composition between the IMM and OMM [28–30]. Since GSH exists in an anionic form at physiological pH [31], the task of GSH entering the mitochondrial matrix is borne by the two anion transporters localized on the IMM, dicarboxylate carrier (DCC), and 2-oxoglutarate carrier (OGC) [32]. DCC exchanges inorganic phosphate, Pi^2^-, or OGC exchanges 2-oxoglutarate (2-OG^2-^) when GSH enters the matrix [33]. These specialties in the IMM make it possible for GSH to transport into the mitochondria. Thus far, the exact mechanism of GSH transporting in mitochondria needs further verification.

#### 2.2.3. Nucleus GSH

In spite of the minimal GSH concentration in the nucleus, studies have confirmed the important role of nuclear GSH in the cell cycle [16, 34, 35]. Cells that are ready for division have higher levels of nuclear GSH [13, 36]. Although there is no definitive proof for this mechanism, it cannot neglect the fact that GSH accumulates in the nucleus at an early stage of cell growth, and when the cells reach confluence, it is reuniformly distributed between the nucleus and the cytosol [34]. The study concerning the correlation between GSH and cell cycle may be helpful for us to better understand cell physiology and cellular metabolic processes.

Lower and medium levels of ROS are generally recognized as inducing mitosis and having beneficial effects in cell growth, while excessive ROS can cause DNA strand breaks, DNA mutations, and DNA double-strand aberrations, further leading to oxidative stress. The sulfhydryl group in GSH is essential in maintaining the status of DNA repair and expression in the nucleus [37]. In the process of ribonucleic acid reduction, GSH acting as a donor of hydrogen catalyzes the reduction of ribonucleic acid to deoxyribonucleic acid, which plays a contributory role in DNA synthesis [38].

#### 2.2.4. ER GSH

ER is interlaced in the cytoplasm and performs a variety of functions, including protein biosynthesis, folding, translocation, and glycosylation and formation of disulfide bonds [39]. The formation of disulfide bonds is the key process for the protein synthesis in ER and also benefits this highly oxidative environment. Accumulation of unfolded or misfolded proteins results in ER stress. The ER glutathione seems to be a special case where the oxidized form accounts for the most. The ratio of GSH : GSSG in ER is as high as 1~15 : 1 [40]. A highly oxidizing environment is a necessary condition for ER to perform its function [41]. Changes in the redox state of ER significantly affect the formation of disulfide bonds in which in this process, GSH is oxidized to GSSG.

### 2.3. GSH Transport

GSH in tissues is mainly derived from hepatocytes, which can only be synthesized in hepatocytes and cannot be degraded. Part of GSH is discharged to the blood through the transport proteins of the hepatocytes, and the other part is discharged to the bile through the bile duct [42, 43].

**Figure 3:**
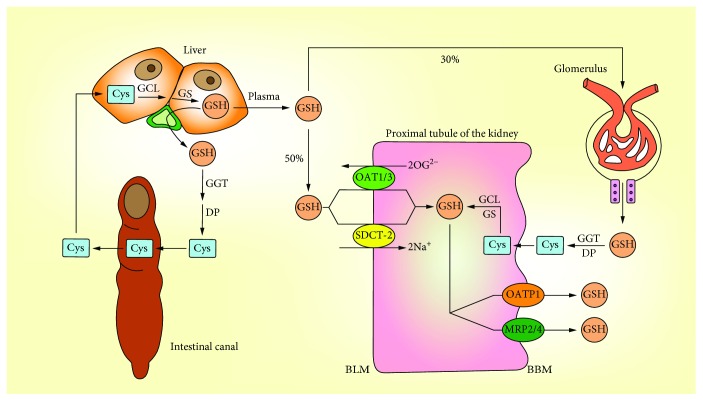
Transport of GSH. The liver is the main source of GSH, the kidney is the main organ that ingests and degrades GSH, and the small intestine participates in the GSH enterohepatic circulation. The renal proximal tubule is the place where the whole process of GSH transport, synthesis, and degradation is completed.

In mammalian tissues, the kidney is the main organ that takes up plasma GSH. 80% of GSH in the plasma is absorbed by the kidney, and 3/8 of them are rapidly decomposed by *γ*-glutamyltransferase (GGT) and dipeptidase (DP) which are located in the brush border membrane (BBM) of the renal tubule after glomerular filtration, and the amino acids absorbed by the renal cells are used to resynthesize proteins or GSH. In addition, the other 5/8 of GSH enters the renal tubule and is absorbed by the specific transporter on the basolateral plasma membrane (BLM) in the form of intact tripeptide [42, 44] (Figure 3). There are two main transporters that facilitate BLM to ingest GSH through a nonfiltering mechanism, and the difference between them is that whether or not they rely on Na^+^ [31]. Organic anion transporter 1 (OAT1) and OAT3 can absorb GSH through exchanging 2-oxoglutarate (2OG). Probenecid and p-aminohippurate (PAH) are two classical inhibitors of OATs that significantly inhibit GSH uptake [45]. Dimethyl succinate (DMS) is a substrate of sodium-dicarboxylate 2 (SDCT2) which significantly inhibits the absorption of GSH by isolated proximal tubule cells [46]. The stoichiometry of Na^+^-GSH cotransportation indicates that at least two Na^+^ couplings are required for absorption per GSH molecule during transport through the SDOT-2 carrier [31].

The process of the GSH outflow in BBM is important for the overall GSH transport. Through the study on the vesicles isolated from the rat kidney cortex, it can be concluded that GSH transport in BBM is a process that is dependent on membrane potential. Unlike GSH transport through BLM, ion coupling is not involved in GSH transport through BBM [47]. Although there is still no evidence to prove the exact vectors that play the direct role in the GSH transport through BBM, it can lead to assumptions based on existing knowledge. There are two types of transporters that contribute to GSH transport [48]. One of the currently more convincing vectors is the organic anion-transporting polypeptide 1 (OATP1), which is expressed in the sinusoidal membrane and demonstrated to transport GSH [49]. Another type of vector that may play a role in the GSH efflux process is multidrug resistance proteins (MRPs) [50]. GSH excreted to the bile is hydrolyzed by GGT and DP on the surface of bile duct epithelial cells or small intestinal epithelial cells. The cysteine produced by hydrolysis can be reused by the small intestine to synthesize GSH and participate in the enterohepatic circulation.

### 2.4. GSH Metabolism

**Figure 4:**
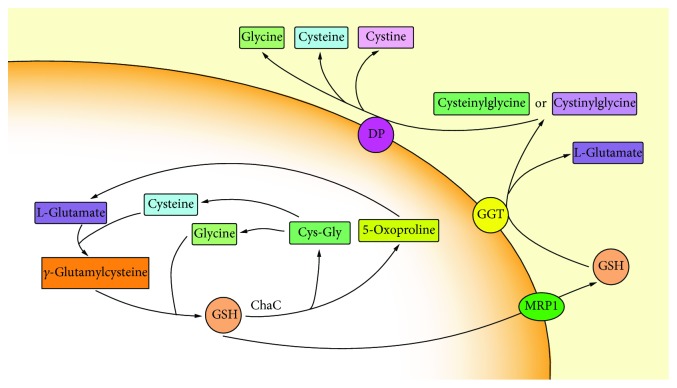
Two degradation pathways of GSH. One way occurs outside the cell where GSH is degraded by *γ*-glutamyltransferase (GGT) which is expressed only on the outer surface of particular cell; the other newly discovered pathway occurs in the cytoplasm where GSH is degraded through ChaC1 and ChaC2.

The structure of GSH is unique in the condensation of glutamate and cysteine producing a *γ*-carboxyl group rather than the usual *α*-carboxyl group. Most enzymes cannot hydrolyze *γ*-carboxyl groups. GGT is the only enzyme expressed on a specific cell surface that is capable of hydrolyzing this particular group [51]. GSH transported by the cell reaches to the GGT active site and is degraded to L-glutamate and cysteinylglycine or cystinylglycine and is then released as glutamate, cysteine, cystine, and glycine under the catalysis of DP. These single amino acids or dipeptides are taken up by the cells to complete the synthesis of GSH (Figure 4).

New pathways for GSH metabolism have also been discovered in recent years. Unlike GGT, the newly discovered ChaC family can enzymatically degrade GSH localized to the cytoplasm [52, 53]. ChaC1 is discovered in bacterial BtrG proteins and mammalian *γ*-GCT proteins, which hydrolyze GSH to produce cysteinyl-free Cys-Gly and 5-oxoproline [54]. It is worth noting that ChaC1 only works on reduced GSH. ChaC2 is another member of the ChaC family, which is found in E. *coli*, yeast, and humans. Its specificity for GSH is similar to that of ChaC1, producing 5-oxoproline and Cys-Gly. Enzyme kinetic studies showed that the catalytic activity of the two was significantly different. The efficiency of the ChaC2 enzyme in degrading GSH was 1/20~1/10 times higher compared to that of the ChaC1 enzyme [55]. GSH metabolism plays a key role in maintaining GSH homeostasis, nutrient recycling and recovery, and signal transduction.

## 3. Antioxidant Role of Cellular GSH

**Figure 5:**
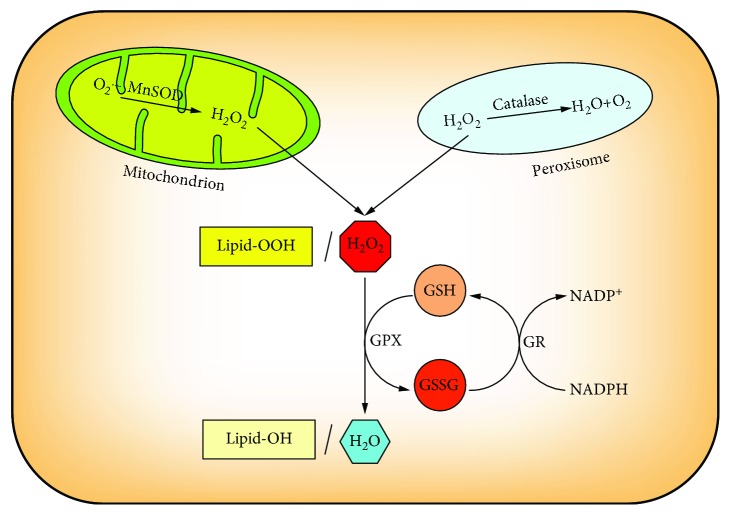
The antioxidant role of cellular GSH. Glutathione peroxidase (GPX) converts H_2_O_2_ and Lipid-OOH to H_2_O and Lipid-OH where GSH is oxidized to GSSG, and glutathione reductase (GR) reduced GSSG to GSH dependent on NADPH, thereby forming a redox cycle to prevent oxidative damage.

ROS is a product of normal cellular metabolism and involved in physiological and biochemical processes. Therefore, balancing the generation and elimination of ROS to maintain the favorable physiological and suitable environment is of great importance [56]. Oxidative stress is caused when the normal oxidation/antioxidant equilibrium state is destroyed. In general, cells are able to cope with mild oxidative stress, while the severe oxidative stress beyond the cell antioxidant capacity can cause damage to lipids, proteins, and DNA, even leading to cell death. There are two main possible strategies to inducing oxidative stress: one is to directly increase the level of ROS and the other is to impair the antioxidant defense system. The GSH system is one of the important antioxidant defense lines against ROS (Figure 5).

Maintenance of cellular redox balance is essential for cell fate. The cellular redox state is often referred to the balance of NAD^+^/NADH, NADP^+^/NADPH, and GSH/GSSG [57]. Among those redox-balancing partners, the two forms of glutathione can be interconverted by enzyme catalysis. Under normal physiological conditions, the vast majority of glutathione is the reduced form. Mitochondria are sites of cellular oxidative respiration, in which ROS are produced by enzymatic or nonenzymatic reactions [58]. Although mGSH accounts for only 10%~15% of the total GSH, its role as an antioxidant cannot be ignored. H_2_O_2_ is a product of aerobic metabolism and is primarily reduced by glutathione peroxidase (GPX) in which in this process, GSH is oxidized to GSSG. GPX is an important peroxide-degrading enzyme. It can catalyze the conversion of GSH to GSSG, reduce toxic peroxides to nontoxic hydroxyl compounds, and promote the decomposition of H_2_O_2_, thereby protecting the structure and function of cell membranes from peroxide interference and damage. GSSG is then reduced to GSH by glutathione reductase (GR) which is associated with NADPH which is oxidized to NADP^+^, thereby forming a redox cycle to prevent oxidative damage [20]. At the same time, GPX reduces lipid peroxides (Lipid-OOH) to nontoxic lipid alcohols (Lipid-OH) with GSH as a substrate. This cycle of mutual transformation enables the continuous elimination of free radicals in the cells [7].

## 4. Role of GSH in Programmed Cell Death

Cancer cells exhibit a higher ROS level and also develop a greater GSH antioxidant system in order to avoid causing oxidative stress. Programmed cell death, including apoptosis, autophagy, necroptosis, and ferroptosis, is initiated by serials of intracellular programs [59]. In some cases, GSH depletion not only triggers one form of programmed cell death but also may initiate multiple forms of cell death. These different forms of cell death may be simultaneously or successively initiated and then interact with each other, and finally, one cell death form may mainly exist [60].

### 4.1. GSH and Apoptosis

**Figure 6:**
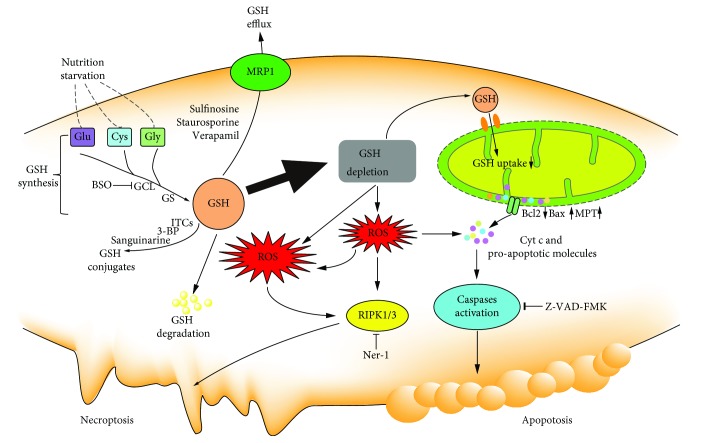
The role of cellular GSH in apoptosis and necroptosis. GSH depletion through nutrition starvation or GSH synthesis inhibition or conjugation with GSH or GSH efflux or GSH degradation induces ROS generation which results in the occurrence of proapoptotic signals, such as the disruption of MMP, increased bax, decreased bcl2, cytochrome c release, and caspase activation. Excess ROS accumulation induces necroptosis.

Apoptosis is the most recognized form of programmed cell death which is initiated and executed by the caspase family. It is a genetically controlled and actively cascading cell death process that is characterized by membrane shrinkage, chromatin condensation, and formation of apoptotic bodies [61]. Studies have shown that the GSH/GSSG redox status is an important indicator of apoptosis in cancer cells. Apoptosis is consistently associated with a reduction in the GSH/GSSG ratio [62]. The decrease in GSH impairs the antioxidant system and leads to the increase in ROS generation which accelerates mitochondrial damage and induces apoptosis (Figure 6).

Intracellular GSH loss precedes the destruction of mitochondrial integrity, cytochrome c release, and caspase activation and is recognized as an early event in the progression of apoptosis in response to different stimuli. GSH depletion occurs in both intrinsic apoptosis and extrinsic apoptosis [63, 64]. A decline in GSH induced ROS generation and the release of cytochrome c, following depletion of the mitochondrial GSH level and caspase 3 activation [65]. Cellular GSH exported into the extracellular space is also demonstrated in the initiation of apoptotic signaling or promotion of apoptotic progression [66]. Cancer cells undergoing apoptosis release a large amount of intracellular GSH into the extracellular environment [67]. Reducing GSH efflux in the apoptotic process could attenuate cell death. Contrarily, stimulation of GSH synthesis could efficiently protect mitochondrial membrane potential loss and inhibit apoptosis [68]. In addition, the exogenous supply with N-acetyl-L-cysteine (NAC) restores the cellular GSH level and prevents the GSH depletion-induced apoptosis [69].

The elevated level of ROS and mGSH/GSSG imbalance can stimulate the intrinsic apoptosis pathway. Impairment of GSH uptake to the mitochondria directly affects the mitochondrial function. Depletion of mGSH leads to the instability of the mitochondrial structure and release of proapoptotic proteins from the outer mitochondrial membrane [70]. The stimuli cause mitochondrial membrane permeabilization through mitochondrial permeability transition (MPT) opening or pores formed by bax and bcl2, resulting in apoptosis-inducing factor release, apoptosome complex formation, and caspase activation [71–73].

### 4.2. GSH and Necroptosis

Although necrosis is originally thought to be a passive and unregulated form of cell death, studies have shown that some form of necrosis can be regulated by intracellular proteins, which is also termed as necroptosis [74, 75]. Necroptosis is an alternative form of programmed cell death with distinct characters in the mitochondria, lysosome, and plasma membrane, exhibiting a translucent cytoplasm, swelling organelles, increased cell volumes, and disruption of the plasma membrane [76, 77]. Necroptosis could be initiated in a way that is similar to extrinsic apoptosis. Receptor-interacting protein kinases 1 (RIPK1) and 3 (RIPK3) are two key regulators involved in the execution of necroptosis. GSH depletion by pharmacological inhibition causes oxidative stress-induced necroptosis [78]. Necrostatin-1, an inhibitor of RIPK1, can protect cell from GSH depletion inducing cell death in HT-22 cells through inhibition on GCL [79]. Artesunate triggers necroptosis by decreasing the GSH/GSSG ratio and increasing ROS generation in human renal carcinoma cells which can be reduced by necrostatin-1 or knockdown of *RIPK1* [80]. To our knowledge, an excess level of ROS induces apoptosis, while massive ROS may lead to necroptosis. GSH depletion-induced ROS generation can simultaneously induce apoptosis and necrosis in cancer cells in some cases (Figure 6). Dimethyl fumarate (DMF) induced typical features of necroptosis-like excessive autophagy, disintegration of mitochondrial membrane potential, LDH release, and accumulation of ROS in colon cancer cells by depleting the cellular GSH level [81]. GSH depletion by cystine starvation or the GSH degradation results in oxidative stress which leads to necroptosis and ferroptosis by directly oxidizing lipids [82].

### 4.3. GSH and Ferroptosis

**Figure 7:**
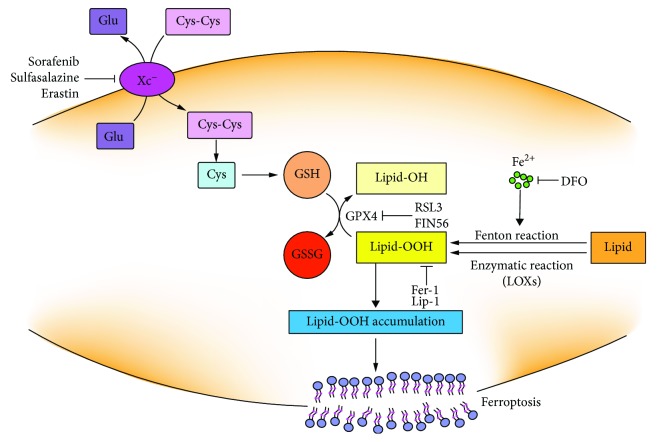
The mechanism of GSH depletion in induction of ferroptosis. Lipid-OOHs can be formed by autoxidation via Fenton reaction or by enzymatic reaction via lipoxygenases (LOXs). Lipid-OOHs are regulated by the balance between the activities of GPX4 and LOXs or Fenton reaction. System X_c_^−^ impairment or GPX4 inactivation leads to Lipid-OOH accumulation which cannot be effectively cleared under the loss of GPX4 activity. Ultimately, the accumulation of Lipid-OOH triggers ferroptosis. Ferroptosis can be inhibited by DFO, liproxstatin-1 (Lip-1), and ferrostatin-1 (Fer-1).

Ferroptosis, a kind of programmed cell death, is morphologically, biochemically, and genetically different from other well-known forms of cell death [83]. The characterized features of ferroptosis are iron dependent, GPX4 inactivation, and lipid ROS accumulation [84]. Ferroptosis can be induced by small molecules or GSH biosynthesis inhibitions or GPX4 impairment or some physiological conditions [85] (Figure 7). Cysteine starvation and further GSH depletion cooperate to elevate lipid ROS. Cystine deprivation induced GSH efflux and extracellular degradation for balancing the intracellular cysteine level [86]. GSH depletion through inhibition on cystine uptake is essential for erastin-induced ferroptosis. Additionally, the knockout of *GCL* could sensitize cells to ferroptosis induced by cysteine starvation [87]. Erastin treatment impairs the antioxidant defenses of the cell by indirectly inactivating GPX4 activity resulting in the increase in the cytoplasmic ROS and lipid ROS accumulation.

GPX4 can convert Lipid-OOH to nontoxic Lipid-OH. GPX4 reduced Lipid-OOH using GSH as a cosubstrate. Pharmacological inhibition or genetical depletion of *GPX4* promotes lipid ROS generation or, what is more, is lethal, while upregulation of *GPX4* can diminish lipid ROS [88–90]. Lipid-OOH formation and membrane damage are sufficient inducers in ferroptosis [91]. RSL3 is identified as a small molecule that enhances the lethality toward oncogene-harboring cancer cells by increasing oxidative stress through altering the iron regulatory proteins and genes [92]. Afterwards, RSL3 is proved to be a ferroptosis inducer by covalently targeting the active site of selenocysteine of GPX4 and resulting in the accumulation of lipid ROS. But the mechanism of RSL3-induced ferroptosis is not by depleting GSH but by inactivating GPX4. *GPX4* silence sensitizes cells to RSL3-induced ferroptosis which is accompanied by lipid ROS accumulation [93]. Consequently, direct inactivation of GPX4 can also induce ferroptotic cell death even when cellular cysteine and GSH levels are normal. FIN 56 is a special inducer of ferroptosis that can cause a slower accumulation of ROS as for the downregulation of GPX4 protein abundance [94]. Together, all these types of small molecules can induce ferroptosis by different modulatory profiles, while ultimately, all of them cause the loss of GPX4 activity and generation of lipid ROS. Therefore, it can conclude that GPX4 is the key regulator of ferroptosis and the GSH antioxidant system plays a central role in the regulation of ferroptosis [90, 95].

Ferroptotic oxidative signals are mainly produced by iron-mediated Fenton reaction or enzymatic reaction via lipoxygenases (LOXs) or when the GSH antioxidant system is impaired [96, 97]. GSH deficiency or GPX4 inactivation in inducing ferroptosis involves the enhanced production of oxygenated phosphatidylethanolamine (PE) species [98]. Suppression on the formation of oxygenated PE species can inhibit ferroptosis [99]. Depletion of GSH through the inhibiting system X_c_^−^ induces ferroptosis that could be prevented by liproxstatin-1 (Lip-1), ferrostatin-1 (Fer-1), and iron chelator deferoxamine (DFO) [83, 100, 101].

### 4.4. GSH and Autophagy

Autophagy is a catabolic process by degrading cytoplasmic constituents or impaired organelles in autolysosomes for recycling under stress condition. Autophagy has long been considered a cell protective mechanism, while excessive autophagy can also trigger cell death and be regarded as a tumor suppressive mechanism [102, 103].

**Figure 8:**
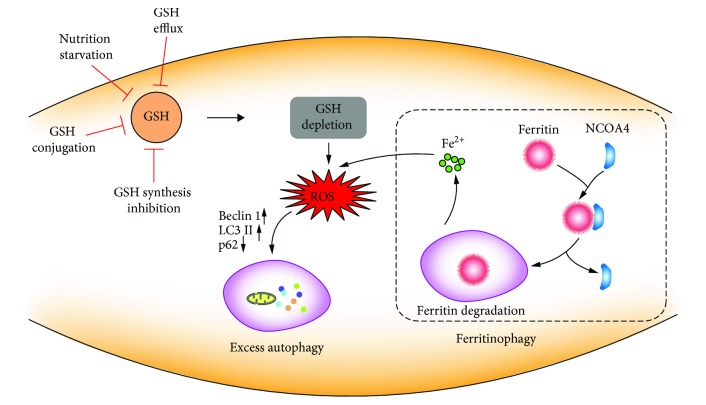
The role of cellular GSH in autophagy. GSH depletion through nutrition starvation or GSH synthesis inhibition or conjugation with GSH or GSH efflux induces ROS generation. ROS accumulation promotes changes in autophagy-related proteins, such as LC3 I/II conversion, p62 degradation, and autophagic vacuole formation. Additionally, ROS induce NCOA4-mediated ferritin degradation in an autophagy process, called ferritinophagy, which is promoting free iron release and accelerating ROS generation.

Growing evidence supports the role of ROS in the regulation of autophagy, but evidence about the mechanism and interplay between GSH and the initiation and promotion of autophagy is still elusive [104]. GSH, one of the principal molecules in the thiol network, has been indicated as the suspect for induction of autophagy [105]. The low level of GSH acts as a signal to activate autophagy as an adaptive stress response [106, 107]. The ways that modulate the intracellular GSH state can drive autophagic response at multiple levels (Figure 8). The dysfunction of system X_c_^−^ by pharmacologic inhibition (sulfasalazine) causes GSH decrease and ROS generation and triggers autophagic cell death [108]. Nutrition starvation can result in the modulation of the cellular GSH content which is mediated by GSH extrusion, GCL inhibition, and the formation of GS-R [109]. Under the GSH depletion case, H_2_O_2_ induced autophagic cell death with increased LC3 conversation and p62 degradation and enhanced autophagic vacuole formation [110]. Together, the decreased cellular GSH level contributes to autophagy and affects the autophagic process. Overall, the possible relationship between GSH and autophagy still deserves to be further investigated.

Ferroptosis is a form of cell death that is dependent on the induction of the autophagic process via a form of cargo-specific autophagy known as ferritinophagy [111]. Autophagy plays a decisive role in the degradation of cytosolic proteins. The impaired autophagic process can induce protein accumulation [112]. The proper function of lysosomes plays an essential role in ferroptotic cell death [113]. The activity of lysosomes is increased in ferroptosis in order to enhance chaperone-mediated autophagy to degrade GPX4 [114]. Inhibition of lysosomal function by bafilomycin A1 (BalfA1) and chloroquine (CQ) can significantly delay the ferroptosis process induced by erastin [115]. Autophagy flux is associated with ferroptosis for promoting the turnover of ferritin in erastin-treated cancer cells [116]. Ferritin degradation is dependent on autophagy where nuclear receptor coactivator 4 (NCOA4) acts as a cargo receptor targeting ferritin to autophagosome [117–119]. Dihydroartemisinin (DHA) induced ferroptosis in acute myeloid leukemia cells through activating the autophagy process with decreased GSH, ferritin degradation, and labile iron accumulation [120]. The exact mechanism of the connection between autophagy and ferroptosis still remains largely unknown.

## 5. GSH Depletion as a Means of Cancer Therapy

A relationship between the increased GSH level and resistance to chemotherapies was observed in many cancers [121]. Impairment in the GSH antioxidant defense system could sensitize cancer cells to current chemotherapeutics. It suggested that the moderate decline in the GSH level would be an effective strategy to improve the sensitivity of cancer cells to chemotherapies. Therefore, depletion of cellular GSH in cancer cells will make them more susceptible and sensitive to oxidative stress and chemotherapies. Cysteine insufficiency or glutamate sufficiency or pharmacological and genetic inhibition of system X_c_^−^ can reduce the resistance of cancer cells to chemotherapies [122]. GSH depletion promotes cancer cell undergoing different forms of programmed cell death, such as apoptosis, necroptosis, autophagy, and ferroptosis. Ways for depleting the cellular GSH level to induce oxidative stress include the following: creation of the source shortage for GSH synthesis, inhibition of the GSH synthesis process, direct conjugation with GSH, and promotion of cellular GSH efflux [123–126].

### 5.1. Inhibition on System X_c_^−^

Cysteine is the main source for protein synthesis. Undoubtedly, it is of critical importance for maintaining the GSH level. Cysteine typically presents in its oxidized form in the extracellular space and can be taken up into the intracellular space via a system X_c_^−^ antiporter. System X_c_^−^, consisting of SLC3A2 (4F2, solute carrier family 3, membrane 2) and SLC7A11 (xCT, solute carrier family 7, membrane 11), forms as a glutamate/cysteine antiporter in the cell membrane [127]. xCT is the light chain of system X_c_^−^. Elevated expression of xCT has been demonstrated in many types of cancer and is related to chemoresistance and poor prognosis in cancer patients [128–133].

A reduction in the uptake of extracellular cysteine can directly cause intracellular GSH depletion. Inhibition on xCT expression triggers cysteine starvation and subsequently induces cell growth arrest in cancer cells. Stabilization of xCT promotes the uptake of cysteine for GSH synthesis and protects cancer cells from high levels of ROS [134]. Therefore, regulation of xCT is considered a promising therapeutic target for cancer therapy [135]. Pharmacological inhibition of system X_c_^−^ inhibits cancer cells *in vitro* and delays tumor growth *in vivo*. Disruption on xCT function inhibits cell invasion and tumor metastasis [136]. The inhibitory effects on cancer cells can be ascribed for the rapid depletion of GSH by xCT dysfunction and subsequently increase in ROS generation.

Erastin is an inhibitor of system X_c_^−^ that can lead to the depletion of GSH [83]. GSH-depleting effects of erastin could be reversed by supplying with GSH and N-acetylcysteine (NAC). Imidazole ketone erastin (IKE), a carbonyl erastin analogue, also exhibits system X_c_^−^ inhibition activity and displays more potency to selective lethality to cancer cells than erastin [137]. Sorafenib promotes ferroptosis in HCC cells by its ability to inhibit system X_c_^−^ and deplete GSH [101]. Sorafenib can also potentiate cisplatin cytotoxicity in resistant head and neck cancer cells through the inhibitory effect on xCT [138]. Sulfasalazine is an anti-inflammatory drug which can be used for the treatment of inflammatory bowel disease and rheumatoid arthritis and is also proved to be a potent inhibitor of system X_c_^−^. It can sensitize cancer cells not only to chemotherapies but also to radiotherapies [139–141]. Pseudolaric acid B, a natural diterpene acid isolated from the root and bark of *Pseudolarix kaempferi*, can trigger ferroptosis in glioma cells by depleting cellular GSH through inhibition of xCT [142, 143].

### 5.2. Inhibition on GCL

*γ*-GCL plays a key role in the synthesis and maintenance of the cellular GSH level. It is the first and rate-limiting enzyme in GSH synthesis consisting of the GCLC catalytic subunit and GCLM modifier subunit [144]. Overexpression of GCL increases the cellular GSH level, and cells exhibit more resistance to oxidative stress [145]. Adrenomedullin induces the expression of *GCLC* and protects cells against oxidative stress [146]. On the contrary, knockdown of *GCLC* could elevate the cellular ROS level [147]. L-Buthionine-(S,R)-sulfoximine (BSO) is an inhibitor of *γ*-GCL. It has been shown to increase the efficacy of nifurtimox against cancer cells and be an effective modulator of GSH-mediated chemoresistance by increasing the *in vitro* cytotoxicity of alkylating agents and radiation [148].

### 5.3. Conjugation with GSH

The most direct strategy to deprive GSH is to react with it. Some natural molecules exhibit good affinity to GSH. Sanguinarine directly reacts with cellular GSH and causes a rapid and sever depletion of GSH. It results in the subsequent modification of the membrane integrity and relates to a promotion of apoptotic response dependent on caspase 3 and caspase 7 activation in PC3 human prostatic adenocarcinoma cells [149]. 3-Bromopyruvate (3-BP), an alkylating agent, has high reactivity toward thiols and rapidly conjugates with GSH in the cell-free system and many cell types [150, 151]. It has been proved to have antitumor activities [152, 153]. Isothiocyanates (ITCs) are natural phytochemicals abundantly existing in cruciferous vegetables. The central carbon of the ITCs is highly electrophilic and can react with thiols. At physiological pH, ITCs react predominantly with the sulfhydryl group of cysteine residues in GSH. Accumulative evidence has proved that ITCs, such as sulforaphane (SFN), phenethyl isothiocyanate (PEITC), and ally isothiocyanate (AITC), are highly effective in chemoprevention and have antitumor activities *in vitro* and *in vivo* [154–158]. PEITC exhibits potential ability against not only solid tumor but also leukemia cells through the rapid deprive of mitochondrial GSH and elevation of ROS [70, 159].

### 5.4. Enhancement of GSH Efflux

The development of the multidrug resistance (MDR) phenotype poses as a major clinical problem that limits the curative potential of anticancer drugs. The characterized phenotype of MDR is the typically increased expressions of P-glycoprotein (P-gp) and MRPs. P-gp and MRPs can extrude anticancer agents out of cell consuming ATP and result in the chemotherapy failure. Inhibition of MRPs could reduce drug resistance in cancer cells, and MRPs act as a potential target in cancer therapy. MRP-1 is identified as a GSSG transporter. Evidence has shown that inhibition on MRP activity promotes the accumulation of GSSG which is cytotoxic to endothelial cell tumors [160]. Sulfinosine has the potential to induce apoptosis and autophagy by decreasing GSH, generating ROS, and inhibiting P-pg and then sensitizes cancer cells to chemotherapies [161]. Modulation of GSH efflux is also a potential strategy to induce cell death in cancers. Staurosporine causes apoptosis in cancer cells associated with exporting cellular GSH [162]. Cancer cells are sensitized to cell death when intracellular GSH is depleted through stimulation of GSH efflux pumps [163]. Natural compound chrysin induces GSH efflux by MRPs to maintain the depleted GSH level and sensitizes cancer cells to chemotherapeutic agents like doxorubicin [164]. Verapamil derivatives can effectively kill cancer cell through leading to apoptosis with the mechanism of stimulating GSH efflux by MRPs [126].

## 6. Conclusions

In this review, accumulative evidence has demonstrated the important role of GSH depletion in the initiation of multiple forms of programmed cell death in cancers and we have highlighted the GSH-based strategies for cancer therapies. As mentioned, some agents trigger not only one type of programmed cell death solely but also multiple forms of cell death simultaneously through altering cellular GSH in cancer cells. While the crosstalks and interrelationships between the multiple forms of cell death induced by GSH modulation in cancer cells are still elusive, the exact death events along with GSH depletion in inducing cell death are still needed to be further explored. In the future work, a better understanding on the mechanism of GSH in triggering different forms of programmed cell death and whether GSH has a role in deciding cell fate will give more implications on the redox-based research concerning cancer therapeutics.
